# Gait training with a wearable powered robot during stroke rehabilitation: a randomized parallel-group trial

**DOI:** 10.1186/s12984-023-01168-x

**Published:** 2023-04-28

**Authors:** Daichi Miyagawa, Akira Matsushima, Yoichi Maruyama, Noriaki Mizukami, Mikio Tetsuya, Minoru Hashimoto, Kunihiro Yoshida

**Affiliations:** 1 Department of Rehabilitation, JA Nagano Koseiren Kakeyu-Misayama Rehabilitation Center Kakeyu Hospital, Ueda, Japan; 2 Department of Neurology, JA Nagano Koseiren Kakeyu-Misayama Rehabilitation Center Kakeyu Hospital, Ueda, Japan; 3 Department of Information Technology, International Professional University of Technology in Tokyo, Tokyo, Japan; 4 AssistMotion Inc, Ueda, Japan; 5 Faculty of Textile Science and Technology, Shinshu University, Ueda, Japan; 6 Division of Neurogenetics, Department of Brain Disease Research, Shinshu University School of Medicine, Matsumoto, Japan

**Keywords:** Robot-assisted gait training, Wearable rehabilitation robot, Stroke rehabilitation, Randomized controlled trial, Hemiparesis

## Abstract

**Background:**

We have developed a wearable rehabilitation robot, “curara®,” and examined its immediate effect in patients with spinocerebellar degeneration and stroke, but its rehabilitative effect has not been clarified. The purpose of this study was to examine the effect of this device on gait training in stroke patients.

**Methods:**

Forty stroke patients were enrolled in this study. The participants were divided randomly into two groups (groups A and B). The participants assigned to group A received RAGT with curara® type 4, whereas those in group B received conventional therapist-assisted gait training. The clinical trial period was 15 days. The participants performed 10 sessions of gait training (5 times per week) each lasting 30 ± 5 min per day. The 10-m walking time (10mWT), and 6-minute walking distance (6MWD) were evaluated as the main outcomes. Timed up and go and Berg Balance Scale (BBS) were also examined. Gait parameters (stride duration and length, standard deviation of stride duration and length, cadence, ratio of the stance/swing phases, minimum/maximum knee joint angle, and minimum/maximum hip joint angle) were measured using a RehaGait®. The items other than BBS were measured on days 0, 7, and 14, whereas BBS was measured on days 0 and 14. The improvement rate was calculated as the difference of values between days 14 and 0 divided by the value on day 0. The improvement rates of the 10mWT and 6MWD were set as the main outcomes.

**Results:**

The data of 35 participants were analyzed. There was no significant difference in the main outcomes between both groups at the end of gait training. As for intragroup changes, gait speed, stride length, stride duration, and cadence were improved significantly between days 0 and 14 in each group. When examining the interaction effect between the day of measurement and group, stride duration (p = 0.006) and cadence (p = 0.012) were more significantly improved in group A than in group B.

**Conclusions:**

This novel wearable powered robot may have the potential to improve gait speed of individuals in stroke rehabilitation.

**Trial registration:**

Japan Registry of Clinical Trials (jRCTs032180163). Registered on February 22, 2019; https://jrct.niph.go.jp/en-latest-detail/jRCTs032180163.

**UMIN Clinical Trials Registry (UMIN000034237):**

Registered on September 22, 2018; https://center6.umin.ac.jp/cgi-open-bin/icdr/ctr_view.cgi?recptno=R000038939.

## Background

Recently, robot-assisted gait training (RAGT) has been applied widely to individuals with stroke to regain and improve walking ability [1]. In the 1990s, body weight-supported treadmill training was introduced in the clinical setting [2]. Currently, rehabilitation robots with different assistive forms for lower limb movements have become popular, e.g., Gait Trainer® and Haptic Walker®, which assist with foot movements, and Lokomat®, which assists with lower limb orthosis on a treadmill. These devices enable the joints of the lower limbs to move in a state close to normal during walking without voluntary efforts from the patient [3–5].

In addition to stationary rehabilitation robots, wearable rehabilitation robots have been developed recently and used widely in gait training, e.g., Hybrid Assistive Limb (HAL®) and ReWALK® [6]. Generally, wearable rehabilitation robots are smaller and lighter than stationary rehabilitation robots; therefore, a great advantage of these devices is that individuals wearing them can move around freely. This makes it possible for people with a wearable rehabilitation robot to perform overground training in daily life. Systematic reviews have reported that rehabilitation robots improve balance and ankle spasticity in patients with brain diseases including stroke [7, 8]. Concerning the rehabilitative effect of HAL®, the degree of walking independence evaluated by the Functional Ambulation Category was significantly improved in the HAL®-wearing group compared to the non-wearing group [9, 10]. In addition, there are reports that RAGT showed more significant improvement in the time up and go (TUG) test, 10mWT and 6MWD than conventional walking training [11, 12].

We started clinical research in 2017 for the practical application of a wearable rehabilitation robot, “curara®”. We have examined the effect of the device in patients with spinocerebellar degeneration [13, 14] and stroke [15]. We have shown that the use of the device improves walking speed, stride length, walking rate, and asymmetry in stroke patients temporarily [15], but we have not yet clarified its rehabilitative effect on gait training. Therefore, the purpose of this study was to examine the effect of the device on gait training in stroke patients.

## Methods

### Participants and instrumentation

A total of 40 individuals participated in this study. They fulfilled all of the inclusion criteria and did not fulfill any of the exclusion criteria. The inclusion criteria were: (1) cerebrovascular disease presenting with hemiplegia; (2) aged ≥ 20 years; (3) 14–90 days after stroke onset; (4) able to walk ≥ 10 m independently with or without a walker and/or brace; and (5) Berg Balance Scale (BBS) score ≥ 26. The exclusion criteria were: (1) possible causes for gait disturbance other than cerebrovascular disease; (2) too thin or obese to fit into the device; and (3) any other reasons that were considered to make the subjects ineligible to participate (for example, severe dementia or psychiatric symptoms, severe spasticity or joint contracture of the paralyzed leg, etc.).

The participants were divided randomly into two groups (groups A and B). The participants in group A were assigned RAGT with the device, whereas those in group B received conventional therapist-assisted gait training. Treatment was randomly assigned at the Data Center of Center for Clinical Research in Shinshu University Hospital. The randomization list has been generated using Viedoc 4 ™ (Pharma Consulting Group, Sweden) that was used as an electronic data capture system for this study. To balance the two treatment arms, dynamic randomization performed between the two treatment arms in a ratio of 1:1 applying the modified algorithm of Pocock and Simon (as implemented in Viedoc). Characteristics of the participants were shown in Table 1. Baseline participants were 65.1 ± 12.9 years [mean ± standard deviation (SD)] in group A and 63.0 ± 12.9 in group B. Brunnstrom Recovery Stage scores were III - VI in Group A and IV - VI in group B. FIM and BBS scores were not different between groups A and B (FIM: 74.8 ± 12.6 vs. 75.6 ± 10.2; BBS: 52.1 ± 4.3 vs. 49.1 ± 6.8, respectively).

**Table 1:** Characteristics of the participants

	Group A (*n* = 17) mean ± SD	Group B (*n* = 18) mean ± SD	*p*-value
**Age (years)**	65.1 ± 12.9	63.0 ± 12.9	0.631
**Height (cm)**	162.2 ± 8.8	166.3 ± 10.0	0.204
**Weight (kg)**	62.2 ± 10.1	66.5 ± 14.0	0.302
**Lower limb length (cm)**	77.3 ± 5.5	79.2 ± 6.0	0.327
**Brunnstrom Recovery Stage** ^**#**^	III-VI	IV-VI	0.916
**FIM score on day 0**	74.8 ± 12.6	75.6 ± 10.2	0.829
**BBS on day 0**	52.1 ± 4.3	49.1 ± 6.8	0.071

BBS: Berg Balance Scale; FIM: Functional Independence Measure; SD: standard deviation

^#^Brunnstrom Recovery Stage for lower legs

We used a wearable “curara® type 4” robot in this study, which weighs approximately 5 kg (four actuator units and the control box) and has a novel structure that does not have direct rigid link between the actuator units on the hip and knee joints. Thus, both the actuator units can function independently. The basic mechanisms of the device are characterized by a torque-sensing technique and synchronized-based control system. Detailed information regarding the device is described in our previous reports [13, 15].

## Outcome measures

The study period was 15 days in total. We evaluated the participants on days 0, 7, and 14. On these days, they were instructed to walk 10 m on a flat floor at a comfortable speed 9 times while wearing a RehaGait® analyzer (HASOMED, Magdeburg, Germany). We measured the 10-m walking time (10mWT) with a stopwatch and the distance walked in 6 min (6-minute walking distance; 6MWD). We also performed timed up and go (TUG) test on days 0, 7, and 14, and BBS on days 0 and 14. We collected the following gait parameters using RehaGait®: stride duration and length, standard deviation of stride duration and length, cadence, ratio of the stance/swing phases, minimum/maximum knee joint angle, and minimum/maximum hip joint angle. We set the improvement rates of the 10mWT and 6MWD as the main outcome measures, which were calculated as the difference of values between days 14 and 0 divided by the value on day 0. All of the measurements shown above were acquired without wearing the device.

## Rehabilitation program

The participants performed 10 sessions of gait training (5 times per week) each lasting 30 ± 5 min session per day. One or two physical therapists accompanied each participant during gait training. For group A, the therapists operated the device and prevented falls, but they did not give any advice on gait to the participants. The participants also received a combination of physical (except gait training), occupational, or speech therapy rehabilitation. The maximum rehabilitation time including gait training was 3 h/day in both groups.

We set synchronization gain, gait cycle, and joint angles as the assist conditions of the device. Among these, synchronization gain was fixed to 0.1 at the hip joint and 0.3 at the knee joint throughout the rehabilitation period in all participants. The gait cycle and joint angles varied from individual to individual, and they were set according to the gait parameters of the fastest gait performance on days 0 and 7. The amplitude of the joint angle was set at 140% at the unaffected hip joint and 110% at the unaffected knee joint, and the gait cycle was set at 85%. Therefore, the assist conditions were set 40% wider hip joint, 10% wider at the knee joint, and 15% faster in the gait cycle than the gait parameters of the fastest gait performance. The conditions set on day 0 were used for gait training on days 1–6, and those updated on day 7 were utilized on days 8–13. These parameters meant that the amount of assistance provided by the robot was larger at the hip joint than at the knee joint, i.e., the device-in-charge robotic support was more influential at the hip joint than at the knee joint. With these assist conditions, the device enabled the participants to reproduce their best gait performance faithfully during gait training.

### Statistical analysis

The distribution of characteristics of the participants between two groups was analyzed by a *t*-test for continuous variables and a chi-square test for categorical data. The differences of the BBS score and TUG test between days 0 and 14 were analyzed by a paired *t*-test. The differences of the gait parameters obtained with RehaGait® were analyzed using a generalized linear mixed model with Bonferroni’s correction. In the model, the day of measurement, group, and the interaction between those two factors were set as the fixed effects, and subject factors and the number of measurements on the same day of measurement were set as random effects. All statistical analyses were performed using IBM SPSS Statistics 24 for Windows. The level of significance was set at *p* < 0.05 in all tests.

## Results

Of the 40 participants, three in group A and one in group B dropped out of the trial; two participants in group A retracted informed consent because of mental health issues and one discontinued the trial due to robot-induced skin problems, while one participant in group B had a second stroke attack during the trial period. A total of 36 participants (17 in group A and 19 in group B) completed the program without any adverse events. Detailed information on the participants is shown in Table 1. However, one participant in group B was excluded from the analysis due to extreme outliers in most of the measured items; therefore, the data of 35 participants were analyzed.

The results of the main outcome measures are shown in Table 2. The mean improvement rates of the 10mWT were 20.6% in group A and 13.9% in group B. However, the difference between both groups was not statistically significant (*p* = 0.099). The mean improvement rates of the 6MWD were 16.8% in group A and 19.4% in group B, again with no significant difference between both groups (*p* = 0.067). The amount of change in the BBS or TUG was not statistically significant between groups A and B.

**Table 2:** Results of the main outcomes, BBS, and TUG

	Group A (*n* = 17) mean ± SD	Group B (*n* = 18) mean ± SD	*p*-value
Improvement rate			
**10mWT (%)**	20.6 ± 11.6	13.9 ± 11.7	0.099
**6MWD (%)**	16.8 ± 18.0	19.4 ± 18.5	0.677
**Amount of change**			
**BBS (score)**	1.3 ± 1.4	2.6 ± 2.7	0.087
**TUG (s)**	-1.4 ± 2.0	-1.3 ± 1.6	0.854

10mWT: 10-m walking time; 6MWD: 6-minute walking distance; BBS: Berg Balance Scale; SD: standard deviation; TUG: timed up and go

The values of the gait parameters obtained by RehaGait® are shown in Table 3. Almost all of the gait parameters, except maximum flexion angles of the hip and knee joints, improved on day 14 in each group. When examining the interaction effect between the day of measurement and group, stride duration (*p* = 0.006) and cadence (*p* = 0.012) were more significantly. Therefore the results improved in group A than in group B. These results were expected to be reflected in gait speed, and the interaction effect of gait speed was very close to the level of statistical significance (*p* = 0.055).

**Table 3:** Gait parameters obtained with RehaGait®

	Group A (*n* = 17)		Group B (*n* = 18)		*p*-value		
	day 0	day 14	day 0	day 14	Intragroup difference^#^		Interaction effect*
					Group A	Group B	
**Stride duration (s)**	1.19 ± 0.17	1.04 ± 0.12	1.15 ± 0.15	1.09 ± 0.19	< 0.001	0.007	**0.006**
**Stride length (m)**	1.07 ± 0.20	1.18 ± 0.22	0.99 ± 0.23	1.08 ± 0.23	< 0.001	< 0.001	0.359
**Gait speed (m/s)**	0.93 ± 0.26	1.17 ± 0.31	0.88 ± 0.28	1.03 ± 0.32	< 0.001	< 0.001	**0.055**
**Cadence (steps/min)**	103.1 ± 14.2	116.6 ± 13.1	105.9 ± 14.4	112.6 ± 17.1	< 0.001	< 0.001	**0.012**
**Ratio of the unaffected** **standing phase (%)**	64.5 ± 3.6	62.8 ± 4.0	63.8 ± 4.0	62.9 ± 3.5	0.004	0.077	0.254
**Ratio of the affected** **standing phase (%)**	66.5 ± 5.0	64.0 ± 3.9	67.0 ± 4.3	65.4 ± 4.3	< 0.001	< 0.001	0.157
**Maximum flexion angles**							
**unaffected knee joint (°)**	55.2 ± 11.4	56.4 ± 9.9	45.6 ± 13.7	47.7 ± 14.8	0.695	0.235	0.449
**affected knee joint (°)**	60.1 ± 7.7	60.6 ± 6.7	53.3 ± 7.4	54.8 ± 7.4	1.000	0.734	0.799
**unaffected hip joint (°)**	33.1 ± 4.7	34.7 ± 5.8	30.1 ± 6.3	31.0 ± 7.4	0.288	0.783	0.553
**affected hip joint (°)**	34.3 ± 4.1	34.7 ± 3.8	32.2 ± 6.8	33.9 ± 7.7	0.957	0.069	0.442
**Symmetry** ^§^	0.98 ± 0.16	1.00 ± 0.17	1.02 ± 0.51	1.01 ± 0.66	1.000	1.000	1.000

^**#**^Intragroup difference between days 0 and 14

*Interaction effect: between the day of measurement and group

^§^Symmetry: ratio of the maximum flexion angles of the affected hip joint to the unaffected hip joint

The index of symmetry was expressed as the ratio of the maximum hip flexion angle of the affected leg to that of the unaffected leg in each group (Table 3). However, there was no significant difference in the index of symmetry within or between the groups.

## Discussion

Seventeen out of 20 participants tolerated and coped well with a 15-day clinical trial with the device without any adverse events. The primary outcome measures in this study showed significant improvements (10–20% of the baseline value) in both groups; however, contrary to our expectations, there were no significant differences between the groups. The same outcome was found for the BBS and TUG.

When analyzing the RehaGait® data, significant intragroup differences in several parameters between days 0 and 14 were found in both groups. In particular, stride duration and cadence showed a more significant improvement in group A compared to the group B (interaction effect: *p* = 0.006 for stride duration, *p* = 0.012 for cadence). As a result, the interaction effect for gait speed almost reached statistical significance (*p* = 0.055). We reported previously that stride duration and cadence are increased in stroke patients when they walked with the device [15]. Thus, the rehabilitative effect of the device on stride duration and cadence was confirmed in the present study. This effect is very likely to contribute to the improvement of walking speed, and the assistance provided by the device, which shortened the gait cycle and enlarged the joint angles, would have led to this favorable change in stride duration and cadence.

In this study, all the participants were rather mild in the severity of hemiparesis at baseline because they had to train with the device (~ 5 kg) burdened when they were randomized to group A. In fact, the averaged gait speed was 0.90 m/sec at baseline (*n* = 36), which was faster than the gait speed (0.08–0.64 m/s) in the participants reported in the meta-analysis study investigating the RAGT effect [16]. In our preliminary study on stroke patients, which was a single-use analysis of curara® type 3, the average walking speed of the subjects was 0.4 m/s (*n* = 15) [15]. In this situation, we have to take the ceiling effect into consideration when interpreting the data, which is one of the major limitations of this study. As we measured the difference between the 10mWT on days 0 and 14, the ceiling effect should be minimized. The actual change in the 10mWT in group A (-0.23 ± 0.12 m/s, mean ± standard deviation) was greater than that in group B (-0.14 ± 0.12 m/s), but the difference between the groups was not statistically significant.

Another concern was whether or not the assistance provided by the device was best for each participant. We did not set synchronization gain at the hip and knee joints according to individual gait performance. Synchronization gain may be related to error learning that involves the trial and error required for motor learning [17]. A lower synchronization gain means a reduced degree of flexibility of intentional joint movement. Ideally the synchronization gain of the device should be set individually, but it is a complicated and time-consuming process to perform during the limited time available for this clinical trial.

During the study, we felt that the affinity of the participants to the robot varied from individual to individual. Roughly speaking, the more elderly individuals tended to have less affinity for the device than the younger ones, and individuals with better walking ability had lower motivation to practice with the robot. One factor influencing affinity to the robot was that RAGT is still being developed as a method for stroke rehabilitation throughout Japan. For RAGT to become more popular, high-quality evidence should be acquired in future randomized case-control studies.

At least, we can say that RAGT with curara® had a rehabilitative effect identical to that of therapist-assisted gait training. Our initial motive was to develop a wearable robot that elderly or handicapped people can use for gait rehabilitation in daily life. The results shown above are encouraging for us to improve the devise further. First, we need to improve its operability and user friendliness so that anybody can use it easily. At the present time, we are creating an advanced type of robot using the experience and knowledge acquired in this study. Furthermore, we need to identify the best application timing and assist conditions for the device in stroke patients.

## Conclusion

RAGT with our novel wearable powered robot may help to improve the walking speed of stroke patients. In order to use the device for walking rehabilitation in daily life, we have to review the selection criteria and outcomes of the subjects in future studies and further improve the operability and usability of the device.

## Data Availability

The datasets used and/or analyzed during the current study are available from the corresponding author on reasonable request.

## List of abbreviations

6MWD: 6-minute walking distance
10mWT: 10-m walking time
BBS: Berg Balance Scale
RAGT: robot-assisted gait training
TUG: timed up and go
